# Common and Specific Associations of Anti-SSA/Ro60 and Anti-Ro52/TRIM21 Antibodies in Systemic Lupus Erythematosus

**DOI:** 10.1155/2013/832789

**Published:** 2013-10-30

**Authors:** Aurora Menéndez, Jesús Gómez, Luis Caminal-Montero, José Bernardino Díaz-López, Iván Cabezas-Rodríguez, Lourdes Mozo

**Affiliations:** ^1^Department of Immunology, Hospital Universitario Central de Asturias, Celestino Villamil S/N, 33006 Oviedo, Spain; ^2^Department of Internal Medicine, Hospital Universitario Central de Asturias, Celestino Villamil S/N, 33006 Oviedo, Spain; ^3^Bone and Mineral Research Unit, Hospital Universitario Central de Asturias, Celestino Villamil S/N, 33006 Oviedo, Spain

## Abstract

Little information exists about the association of anti-SSA/Ro60 and anti-Ro52/TRIM21 with systemic lupus erytematosus (SLE) features. In this work, we analysed the associations of both anti-Ro reactivities with clinical and immunological manifestations in 141 SLE patients. Photosensitivity and xerophtalmia/xerostomia were found to be positively associated with both anti-SSA/Ro60 (*P* = 0.024 and *P* = 0.019, resp.) and anti-Ro52/TRIM21 (*P* = 0.026 and *P* = 0.022, resp.). In contrast, a negative association was detected regarding anti-phospholipid antibodies, anti-SSA/Ro60 having a stronger effect (*P* = 0.014) than anti-Ro52/TRIM21. Anti-SSA/Ro60 showed a specific positive association with hypocomplementemia (*P* = 0.041), mainly with low C4 levels (*P* = 0.008), whereas anti-Ro52/TRIM21 was found to be positively associated with Raynaud's phenomenon (*P* = 0.026) and cytopenia (*P* = 0.048) and negatively associated with anti-dsDNA (*P* = 0.013). Lymphocytes are involved in the relationship between anti-Ro52/TRIM21 and cytopenia since positive patients showed lower cell levels than negative patients (*P* = 0.036). In conclusion, anti-SSA/Ro60 and anti-Ro52/TRIM21 showed both common and specific associations in SLE. These data thus increase evidence of the different associations of the two anti-Ro specificities even in a particular disease.

## 1. Introduction

Anti-SSA/Ro60 and anti-Ro52/TRIM21 are among the most commonly detected autoantibodies in the routine screening for systemic autoimmune diseases. Although both antibody reactivities were considered to form part of the anti-Ro system for a long time, now it is clearly established that their antigens are different, do not form part of a stable macromolecular complex, and are located in different cellular compartments (reviewed in [1]). Moreover, anti-SSA/Ro60 and anti-Ro52/TRIM21 antibodies have also been associated with a different pattern of clinical manifestations. Thus, the presence anti-SSA/Ro60 is related to autoimmune processes, mainly systemic lupus erythematosus (SLE) and Sjögren's syndrome (SS), whereas anti-Ro52/TRIM21 shows a wider spectrum of disease associations [1–7]. The main clinical autoimmune entities associated with anti-Ro52/TRIM21 are SS, systemic sclerosis (SSc), liver autoimmune diseases, and, specially, myositis where it has been considered as an independent marker [4, 6–14]. Also, anti-Ro52/TRIM21 has been detected in nonautoimmune conditions such as infections and neoplastic diseases [7–9]. Furthermore, different associations with specific clinical manifestations have also been reported especially for anti-Ro52/TRIM21. Indeed, this anti-Ro reactivity is strongly associated with congenital heart block in neonatal lupus and with interstitial lung disease [9, 10, 15]. Anti-Ro52/TRIM21 has also been related to a more severe disease in SS, myositis, primary biliary cirrhosis, and autoimmune hepatitis [11, 16–18].

Among systemic autoimmune diseases, SLE displays a specific anti-Ro antibody pattern. Thus, although simultaneous reactivity is the more frequent antibody pattern, this disease shows the highest prevalence of isolated anti-SSA/Ro60 [2, 4, 6, 7]. In SLE, antibodies against the Ro system have been historically associated with photosensitivity, but little information exists about the association of both anti-Ro reactivities with other clinical manifestations [19–21]. Therefore, with this background in mind, the aim of this work was to analyse if anti-SSA/Ro60 and anti-Ro52/TRIM21 antibodies are differentially associated with the clinical classification criteria and other frequent manifestations of SLE. 

## 2. Patients and Methods 

### 2.1. Patients, Sera Selection, and Analyzed Features

Sera from 141 SLE patients (131 females, mean age at diagnosis 36.7 ± 14.5 years) who fulfilled the American College of Rheumatology (ACR) criteria were selected for this study [22]. These patients were followed up at the Internal Medicine Autoimmune Disease Unit, Hospital Universitario Central de Asturias, and their clinical and immunologic features were recorded in a database of SLE patients established in our region from 2004 which is periodically updated [23]. Features recorded in this database included the ACR classification criteria and other related SLE manifestations or immunological parameters. In this work, all the features except cytopenia were cumulatively registered. Cytopenia was considered at diagnosis in order to avoid the influence of treatment on the haematological parameters. Only those features whose prevalence was higher than 10% were statistically analysed. In particular, the features included in the analysis were the ACR classification criteria, nonscarring alopecia, xerophthalmia/xerostomia, Raynaud's phenomenon, and hypocomplementemia. All classification criteria were defined as indicated in the 1996 ACR criteria with the exception of neurologic disorders. In this SLE manifestation, organic brain syndrome, visual disturbances, and peripheral and cranial nerve disease were also considered beside seizures and psychosis. Hypocomplementemia was defined as having low C3 and/or C4 levels (<0.8 mg/dL and <0.15 mg/dL, resp.). 

Sera corresponding to different patient's revisions were collected and stored at −20°C. The last serum from each patient was selected for the study (period of collection from February 2007 to March 2011). The mean age at time of analysis was 47.8 ± 14.7 years. 

### 2.2. Determination of Autoantibodies, Complement, and Haematological Parameters

Determination of anti-SSA/Ro60 and anti-Ro52/TRIM21 antibodies in the 141 selected SLE patients was performed by fluoro enzyme immunoassay (Thermo Fisher Scientific-Phadia GmbH, Freiburg, Germany). The assay was carried out on an automated ImmunoCAP 250 analyser. In all patients, other SLE ANA specificities (anti-dsDNA, SS-B/La, U1RNP, and Sm) and anticardiolipin (CL) IgG and IgM antibodies were also simultaneously determined with the same methodology. C3 and C4 levels were measured by nephelometry (Beckman Coulter Inc., California, USA). 

Levels of haematologic parameters, quantified using an Advia 2120 analyzer (Siemens Healthcare, Erlangen, Germany), were available at the time of autoantibody determination in 128 patients (119 females; mean age at diagnosis and at analysis 36.7 ± 14.8 and 47.9 ± 14.5 years, resp.). Leucopenia, lymphopenia, and thrombocytopenia were considered according to the ACR criteria (<4.00 × 10^3^/*μ*L, <1.50 × 10^3^/*μ*L, and <100.00 × 10^3^/*μ*L, resp.) and anaemia was defined by a haemoglobin concentration of <11.0 mg/dL in women and <12.0 mg/dL in men. 

### 2.3. Statistical Analysis

The association of the presence of anti-SSA/Ro60 and anti-Ro52/TRIM21 with SLE manifestations was analyzed by binary logistic regression adjusted for sex and age at diagnosis and at time of analysis. Previously, the presence of both anti-Ro specificities and of anti-dsDNA, SSB/La, RNP, Sm, and CL antibodies was simultaneously analyzed by forward stepwise logistic regression procedure in order to show its influence on each clinical manifestation and immunological parameter. When appropriate, other autoantibodies found to be statistically influential were included in the binary logistic regression analysis of anti-SSA/Ro60 and anti-Ro52/TRIM21 associations. In this analysis, both anti-Ro reactivities were first included together as independent variables. In order to avoid multicollinearity effects caused by the close relationship existing between both specificities, an antibody separate analysis was performed when a not statistically significant association was found. Odds ratios (OR) and their 95% confidence interval (CI) were computed. Levels of complement and haematological parameters were compared by Student's *t*- or Mann-Whitney *U* tests according to data distribution. Results were considered statistically significant when the *P* value was <0.05. Statistical analysis was performed using SPSS 15.0 statistical software package (SPSS Inc., Chicago, IL, USA).

## 3. Results

### 3.1. Prevalence of Anti-SSA/Ro60 and Anti-Ro52/TRIM21 in SLE Patients

The presence of anti-SSA/Ro60 and/or anti-Ro52/TRIM21 antibodies was detected in 62 out of the 141 SLE patients analyzed (44.0%). Simultaneous reactivity was the main observed antibody pattern, 37 patients being positive for both antibodies (26.2%). Anti-SSA/Ro60 alone was present in 23 (16.3%), whereas isolated anti-Ro52/TRIM21 reactivity was only found in two patients (1.4%). Thus, the antibody pattern in this patients' series was in agreement with the established relationship between anti-SSA/Ro60 and SLE and it was not associated with either age at diagnosis or gender prevalence (Table 1).

### 3.2. Associations of Anti-SSA/Ro60 and Anti-Ro52/TRIM21 with Clinical Manifestations in SLE

When anti-SSA/Ro60 and anti-Ro52/TRIM21 were simultaneously included as independent variables in the regression analysis, the only significant association found was cytopenia with anti-Ro52/TRIM21 (Table 2). Due to this finding, we additionally analyzed the relationship between these autoantibodies and the different types of haematologic ACR criteria, and a trend towards an association between anti-Ro52/TRIM21 and leucopenia/lymphopenia was observed (Table 2). Thrombocytopenia could not be analyzed since all thrombocytopenic patients with anti-Ro antibodies were positive for both specificities (4 out of 4) neither was haemolytic anaemia analyzed due to the low number of patients (only five) with this blood disorder. 

Similarly to cytopenia, oral ulcers and Raynaud's phenomenon were also found to be positively, although not significantly, associated with anti-Ro52/TRIM21 (Table 2). When anti-Ro52/TRIM21 was separately analyzed without including anti-SSA/Ro60 as other independent variable, only the association with Raynaud's phenomenon became statistically significant (OR 2.46, CI 95% 1.11–5.43, *P* = 0.026). 

In contrast, photosensitivity and xerophthalmia/xerostomia showed a not statistically significant positive association with both anti-SSA/Ro60 and anti-Ro52/TRIM21 (Table 2). This lack of significance could be due to the existing close relation between both anti-Ro specificities. Thus, as it could be expected, when anti-SSA/Ro60 and anti-Ro52/TRIM21 were separately analysed, the association became statistically significant both for photosensitivity (OR 2.35, CI 95% 1.12–4.96, *P* = 0.024 and OR 2.75, CI 95% 1.13–6.71, *P* = 0.026 for, resp., anti-SSA/Ro60 and anti-Ro52/TRIM21) and xerophthalmia/xerostomia (OR 2.57, CI 95% 1.16–5.68, *P* = 0.019 and OR 2.62, CI 95% 1.15–5.99, *P* = 0.022 for, resp., anti-SSA/Ro60 and anti-Ro52/TRIM21). 

None of the remaining clinical manifestations showed a significant association with anti-SSA/Ro60 and/or anti-Ro52/TRIM21 whether both antibodies were simultaneously or separately analyzed. 

### 3.3. Associations of Anti-SSA/Ro60 and Anti-Ro52/TRIM21 with Immunological Parameters in SLE

Anti-SSA/Ro60 and anti-Ro52/TRIM21 also showed a different association with SLE- related immunological parameters. Indeed, when the two anti-Ro reactivities were considered together as independent variables, an opposite behavior was observed regarding the presence of anti-dsDNA antibodies and hypocomplementemia, anti-SSA/Ro60 being positively associated and anti-Ro52/TRIM21 negatively associated (Table 3). Nevertheless, the only associations found to be statistically significant were the negative relationship between anti-Ro52/TRIM21 and anti-dsDNA antibodies and the positive one between anti-SSA/Ro60, and hypocomplementemia. Both low C3 and C4 levels were found to be positively associated with anti-SSA/Ro60 but only the association with C4 was statistically significant. Furthermore, levels of this complement fraction, but not those of C3, were also significantly lower in anti-SSA/Ro60 positive patients than in negative patients at the time of autoantibody determination (0.16 ± 0.06 mg/dL versus 0.19 ± 0.07 mg/dL, *P* = 0.010 and 0.92 ± 0.30 mg/dL versus 0.96 ± 0.29 mg/dL, *P* = 0.397 for, resp., C4 and C3).

On the other hand, antiphospholipid antibodies were found to be negatively associated with anti-SSA/Ro60 (Table 3). Although both anti-Ro positive groups showed a similar decrease in the percentage of patients with antiphospholipid antibodies, the OR obtained in the anti-Ro52/TRIM21 group was nearly 1 (0.97), whereas that corresponding to the group of anti-SSA/Ro60 positive patients was 0.33. This finding probably reflected a stronger involvement of the coexisting anti-SSA/Ro60 reactivity in the negative relationship with antiphospholipid antibodies. In fact, a negative statistically significant association was only found for anti-SSA/Ro60 in the separate analysis of both specificities (OR 0.32, CI 95% 0.13–0.79, *P* = 0.014 and OR 0.42, CI 95% 0.15–1.20, *P* = 0.104 for anti-SSA/Ro60 and anti-Ro52/TRIM21 resp.). Among the analysed antiphospholipid antibodies, anti-CL IgG/IgM and lupus anticoagulant (LA) were those found to be most involved in this negative relationship (Table 3). Consistently with that observed when analyzing the whole antiphospholipid group, only anti-SSA/Ro60 was found to be statistically significant associated with anti-CL IgG/IgM when the two anti-Ro antibodies were separately analysed (OR 0.29, CI 95% 0.09–0.92, *P* = 0.036 and OR 0.28, CI 95% 0.06–1.27, *P* = 0.098 for anti-SSA/Ro60 and anti-Ro52/TRIM21 resp.). The association with LA was not statistically analyzed due to the very low number of positive patients with anti-SS-Ro60 (2 patients) or Ro52/TRIM21 antibodies (1 patient only). 

Anti-Sm was the only analysed immunological parameter found not be associated either with anti-SSA/Ro60 or anti-Ro52/TRIM21 (Table 3).

### 3.4. Differential Association of Anti-SSA/Ro60 and Anti-Ro52/TRIM21 with Haematological Parameters

In order to confirm the previously observed association between anti-Ro52/TRIM21 and cytopenia, we compared the levels of haematological parameters at time of analysis in 128 SLE patients on the basis of their anti-Ro52/TRIM21 status (Table 4). Out of these patients, 110 (86.6%) were treated with antimalarials, 48 (37.8%) with corticosteroids, and 30 (23.6%) with immunosuppressive drugs (azathioprine, methotrexate, or mycophenolate mofetil). None was receiving biological therapy. Mean leukocyte levels were found to be significantly lower in the group of patients with anti-Ro52/TRIM21 antibodies (*P* = 0.049). This effect was mainly exerted on lymphocytes since anti-Ro52/TRIM21 positive patients showed significantly lower lymphocyte levels than negative patients (*P* = 0.036). Moreover, the association between anti-Ro52/TRIM21 and lymphopenia was further confirmed by logistic regression adjusted for sex, age, and treatment at time of analysis (OR 2.72, 95% CI 1.08–6.88, *P* = 0.035). 

In contrast, when considering anti-SSA/Ro60, the levels of haematological parameters showed a different behaviour (Table 5). In fact, no statistically significant differences were found regarding any leukocyte population whereas anti-SSA/Ro60 positive patients had significantly lower haemoglobin levels than negative patients (*P* = 0.036). In spite of this significant difference, mean haemoglobin level in positive patients was within the normal range. On the other hand, platelet levels were almost significantly higher in anti-SSA/Ro60 positive patients than in negative patients (*P* = 0.052). This finding could be influenced by the negative association found by us between this anti-Ro specificity and anti-CL IgG/IgM antibodies which, in turn, are known to diminish the platelet levels. Indeed, positive patients for anti-CL IgG/IgM showed a trend to have lower platelet levels than negative patients (209.8 ± 69.3 × 10^3^/*μ*L versus 241.1 ± 69.6 × 10^3^/*μ*L, *P* = 0.079, resp.).

## 4. Discussion

In this work, anti-SSA/Ro60 and anti-Ro52/TRIM21 have been shown to display a different pattern of clinical and immunological associations in SLE. According to the previously described relationship between anti-SSA/Ro60 and SLE [2, 4, 6, 7], this anti-Ro specificity was found to be positively associated with hypocomplementemia, an SLE- related immunological feature. On the other hand, a negative association was observed between anti-Ro52/TRIM21 and anti-dsDNA which is consistent with the higher prevalence of this antibody in other autoimmune conditions such as SS, myositis, SSc, and liver diseases [1, 4, 6–14]. 

Both anti-Ro reactivities also displayed different behaviour regarding haematologic abnormalities. Thus, anti-Ro52/TRIM21 was found to be significantly associated with lymphopenia, independently of the therapeutic regime, whereas patients with anti-SSA/Ro60 antibodies showed higher platelet numbers and lower haemoglobin levels than negative patients. Lymphopenia has before been related to the presence of anti-Ro antibodies in SLE and SS [24–28]. Our findings support the specific relationship of anti-Ro52/TRIM21 with lymphopenia in SLE described in previous works [29, 30]. Among the different lymphocyte subsets, evidence exists that CD4+ and NK cells are involved in the lymphopenia associated with anti-Ro or, specifically, with anti-Ro52/TRIM21 [27, 29]. The Ro52/TRIM21 antigen is a cytoplasmic protein that can be induced and redistributed to the nucleus or the cell surface by several stress and proinflammatory stimuli, such as type I and II IFNs, in different cell types including lymphocytes [31–33]. Interestingly, Ro52/TRIM21 expression has been shown to be upregulated in peripheral blood mononuclear cells of SLE and SS patients [34]. It is tempting to speculate that a proinflammatory environment characteristic of systemic autoimmune diseases could induce the expression of Ro52/TRIM21 on the lymphocyte surface thus allowing the binding of specific antibodies that would finally cause cell death. In this regard, it has been described that autologous sera containing anti-Ro increase cell death by apoptosis in SLE lymphocyte cultures [35]. 

Anti-Ro antibodies have previously been found to be associated with a lower prevalence of thrombocytopenia in SLE patients [36]. A possible role of the anti-SSA/Ro60 reactivity in this negative association is supported by the trend towards higher levels of platelet levels in anti-SSA/Ro60 positive patients than in negative patients observed by us. Nevertheless, our data suggest that this protective effect would be indirect and inversely dependent on the antiphospholipid antibody status. In fact, we have found that anti-SSA/Ro60 was negatively associated with antiphospholipid and anti-CL IgG/IgM antibodies which, in turn, have been related to low platelet levels [37, 38]. Our data also support a higher involvement of the anti-SSA/Ro60 specificity in the previously pointed out negative association of anti-Ro with anti-CL antibodies [39]. Finally, the observed negative effect of anti-SSA/Ro60 on haemoglobin levels is not sufficient enough to be considered as anaemia. Thus, all these findings suggest that anti-SSA/Ro60 has no pathological effect on any haematological population in contrast to that observed for anti-Ro52/TRIM21.

Similar to that observed in relation to lymphopenia, anti-Ro52/TRIM21 but not anti-SSA/Ro60 was found to be positively associated with Raynaud's phenomenon. This relationship was independent of the presence of anti-U1RNP, an antibody known to be related to Raynaud's phenomenon even in SLE [21, 36]. Antibodies against the Ro system have also been found to be associated with Raynaud's phenomenon in SS [40]. Supporting the specific role of anti-Ro52/TRIM21 in this association, it is interesting to note that this antibody has been detected in approximately 20% of patients with SSc, where Raynaud's phenomenon is a cardinal feature [41]. Furthermore, anti-Ro52/TRIM21 was also reported to be the second most common autoantibody in one SSc cohort [10]. 

Photosensitivity and xerophthalmia/xerostomia were the only features found to be positively associated with both anti-SSA/Ro60 and anti-Ro52/TRIM21. These associations were only statistically significant when both autoantibodies were separately analyzed probably due to the close relationship existing between them. Although antibodies against the Ro system have been historically considered to be associated with photosensitivity, this relationship remains controversial [19, 20, 36, 42]. In addition, little is known about the association with the two different reactivities [21]. Nevertheless, enhanced expression of SSA/Ro60 and Ro52/TRIM21 has been observed in the cytoplasm or cell surface of keratinocytes after UV irradiation thus supporting the relationship between photosensitivity and reactivity against both antigens [43, 44].

On the other hand, the association between xerophthalmia/xerostomia and anti-Ro is clearly established in SLE patients [36, 45]. Similar to our findings, an association with either anti-SSA/Ro60 or anti-Ro52/TRIM21 has also been reported [21]. These two Ro antigens have been reported to be expressed in the cell surface of human ductal epithelial cells during apoptosis thus suggesting a role of this cell death mechanism in the induction of anti-SSA/Ro60 and anti-Ro52/TRIM21 responses [46]. Furthermore, specific anti-SSA/Ro60 and anti-Ro52/TRIM21 B cells with a differentiating pattern compatible with plasma cells have been detected in salivary glands of SS patients [47]. 

## 5. Conclusions

Anti-SSA/Ro60 and anti-Ro52/TRIM21 showed a different pattern of clinical and immunological associations in SLE. Beside common positive associations with photosensitivity and xerophthalmia/xerostomia, the two anti-Ro reactivities showed both positive and negative specific associations. Anti-SSA/Ro60 was found to be positively associated with hypocomplementemia but negatively with antiphospholipid antibodies, whereas anti-Ro52/TRIM21 showed a positive association with lymphopenia and Raynaud's phenomenon and a negative relationship with anti-dsDNA antibodies. Thus, our data increases evidence on the different associations of both anti-Ro specificities with specific clinical manifestations even in a single disease.

## Figures and Tables

**Table 1:** Demographic characteristics of SLE patients on the basis of their anti-SSA/Ro60 and anti-Ro52/TRIM21 pattern.

Antibody pattern			Age at SLE diagnosis	Gender
Anti-SSA/Ro60	Anti-Ro52/TRIM21	141 patients *n* (%)	Mean ± SD years	Female (%)
+	+	37 (26.2)	38.8 ± 12.5	94.6
+	−	23 (16.3)	35.9 ± 13.8	91.3
−	+	2 (1.4)	32.5 ± 10.6	100
−	−	79 (56.0)	36.1 ± 15.8	92.4

**Table 2:** Association of anti-SSA/Ro60 and anti-Ro52/TRIM21 with clinical manifestations in SLE.

	141 patients		Anti-SSA/Ro60 (*n* = 60)			Anti-Ro52/TRIM21 (*n* = 39)		
Clinical manifestations	*n*	%	*n*	%	OR (95% CI)	*n*	%	OR (95% CI)
Malar rash	88	62.4	38	63.3	0.82 (0.33–2.08)	26	66.7	1.68 (0.59–4.76)
Discoid lesions	29	20.6	13	21.7	1.13 (0.38–3.39)	7	17.9	0.69 (0.20–2.41)
Photosensitivity^a^	89	63.1	45	75.0	1.66 (0.64–4.29)	31	79.5	1.90 (0.62–5.87)
Oral ulcers	78	55.3	31	51.7	0.45 (0.17–1.16)	24	61.5	2.54 (0.88–7.38)
Nonscarring alopecia^b^	75	53.2	32	53.3	0.58 (0.21–1.56)	23	59.0	1.09 (0.35–3.73)
Arthritis^c^	118	83.7	49	81.7	1.50 (0.37–6.09)	31	79.5	0.98 (0.22–4.33)
Serositis	28	19.8	12	20.0	1.11 (0.37–3.37)	7	17.9	0.88 (0.25–3.12)
Renal involvement^d^	45	31.9	21	35.0	1.21 (0.45–3.29)	13	33.3	1.33 (0.44–4.06)
Neurologic disorder^e^	23	16.3	11	18.3	1.60 (0.47–5.42)	7	17.9	0.95 (0.25–3.58)
Xerophthalmia/xerostomia	37	26.2	22	36.7	1.86 (0.65–5.33)	16	41.0	1.69 (0.56–5.05)
Raynaud's phenomenon^f^	67	47.5	33	55.0	1.04 (0.41–2.61)	24	61.5	2.42 (0.86–6.84)
Neurologic disorder	23	16.3	11	18.3	1.31 (0.41–4.25)	7	17.9	0.98 (0.27–3.58)
Cytopenia	88	62.4	39	66.7	0.67 (0.27–1.66)	29	74.3	**2.92 (1.01–8.48)** ^ g^
Leukopenia or lymphopenia	77	54.6	36	60.0	1.08 (0.44–2.64)	25	64.1	1.74 (0.63–4.78)
Thrombocytopenia^h^	18	12.8	4	6.7		4	10.2	
Haemolytic anaemia^h^	5	3.5	2	3.3		3	7.7	

Anti-SSA/Ro60 and anti-Ro52/TRIM21 analyzed together as independent variables by binary logistic regression analysis adjusted for sex and age (at diagnosis and time of analysis). Other autoantibodies also included as independent variable after selection by forward step procedure: ^a^anti-dsDNA (negative association), ^b^anti-SS-B (positive association), ^c^anti-SSB (negative association), ^d^anti-dsDNA (positive association), ^e^anti-cardiolipin (positive association), ^f^anti-RNP (positive association), ^g^ *P* = 0.048, and ^h^not statistically analysed.

**Table 3:** Association of anti-SSA/Ro60 and anti-Ro52/TRIM21 with immunological parameters in SLE.

Immunological parameters	141 patients		Anti-SSA/Ro60 (*n* = 60)			Anti-Ro52/TRIM21 (*n* = 39)		
	*n*	%	*n*	%	OR (95% CI)	*n*	%	OR (95% CI)
Anti-dsDNA	102	72.3	45	75.0	1.93 (0.64–5.79)	26	66.7	**0.23 (0.07–0.73)** ^ b^
Anti-Sm	15	10.6	7	11.6	1.05 (0.24–4.61)	5	12.8	1.2 (0.25–5.86)
Antiphospholipid	34	24.1	8	13.3	0.33 (0.10–1.08)	5	12.8	0.97 (0.24–3.94)
Anticardiolipin IgG/IgM	20	14.2	4	6.7	0.38 (0.09–1.65)	2	5.1	0.57 (0.08–3.88)
Anti-*β*_2_glicoprotein IgG/IgM	20	14.2	6	10.0	0.52 (0.13–2.08)	4	10.2	1.07 (0.22–5.27)
Lupus anticoagulant^e^	14	9.9	2	3.3		1	2.5	
Low complement^a^	106	75.2	48	80.0	**4.61 (1.07–19.97)** ^ c^	28	71.8	0.24 (0.05–1.09)
Low C3^a^	89	63.1	41	68.3	2.44 (0.78–7.64)	24	61.5	0.52 (0.15–1.79)
Low C4^a^	97	68.8	47	78.3	**6.63 (1.63–26.94)** ^ d^	27	69.2	0.25 (0.06–1.08)

Anti-SSA/Ro60 and anti-Ro52/TRIM21 analyzed together as independent variables by binary logistic regression analysis adjusted for sex and age (at diagnosis and time of analysis). Other autoantibodies also included as independent variable after selection by forward step procedure: ^a^anti-dsDNA (positive association), ^b^ *P* = 0.013, ^c^ *P* = 0.041, ^d^ *P* = 0.008, and ^e^not statistically analysed.

**Table 4:** Haematological parameters according to the anti-Ro52/TRIM21 status.

	Anti-Ro52/TRIM21		
	Positive *n* = 33	Negative *n* = 95	
	Mean ± SD or median (range)*	Mean ± SD or median (range)*	*P* value
Leucocytes ×10^3^/*µ*L	5.11 ± 1.37	5.94 ± 2.25	**0.049**
Lymphocytes ×10^3^/*µ*L	1.27 ± 0.48	1.52 ± 0.61	**0.036**
Neutrophils ×10^3^/*µ*L	2.94 (1.70–6.90)	3.38 (1.00–10.46)	0.247
Platelets ×10^3^/*µ*L	241.24 ± 78.23	235.19 ± 67.53	0.179
Haemoglobin mg/dL	12.42 ± 1.53	12.91 ± 1.38	0.089

*According to data distribution.

**Table 5:** Haematological parameters according to the anti-SSA/Ro60 status.

	Anti-SSA/Ro60		
	Positive *n* = 52	Negative *n* = 76	
	Mean ± SD or median (range)*	Mean ± SD or median (range)*	*P* value
Leucocytes ×10^3^/*µ*L	5.64 ± 2.08	5.78 ± 2.10	0.704
Lymphocytes ×10^3^/*µ*L	1.35 ± 0.48	1.52 ± 0.64	0.098
Neutrophils ×10^3^/*µ*L	3.26 (1.60–10.46)	3.24 (1.00–10.13)	0.821
Platelets ×10^3^/*µ*L	251.29 ± 73.33	224.50 ± 66.59	0.052
Haemoglobin mg/dL	12.46 ± 1.41	13.00 ± 1.41	**0.036**

*According to data distribution.
